# Antifatigue and antiaging effects of Chinese rice wine in mice

**DOI:** 10.1002/fsn3.830

**Published:** 2018-10-18

**Authors:** Pan Zhao, Jing Wang, Wei Zhao, Xiaoli Ma, Haiji Sun

**Affiliations:** ^1^ Key Laboratory of Animal Resistance Biology of Shandong Province School of Life Science Shandong Normal University Jinan China; ^2^ Institute of Millet Crops Hebei Academy of Agriculture and Forestry Sciences Jiazhuang Shi China; ^3^ Central Laboratory Jinan Central Hospital Affiliated to Shandong University Jinan China

**Keywords:** antiaging effect, antifatigue activity, antioxidant activity, Chinese rice wine

## Abstract

Chinese rice wine (CRW) is widely known for keeping good health and commonly used in the traditional Chinese medicine prescription guiding drug. This study assesses the effects of CRW on antifatigue and antiaging activities in mice models. Mice were randomly divided into four groups performing with 0.25 ml of distilled water, 0.15, 0.25, and 0.4 ml of CRW for 15 consecutive days. The CRW could obviously increase the content of liver glycogen (LG) and decrease the levels of blood lactic acid (BLA)and blood urea nitrogen (BUN) to improve antifatigue ability. In antiaging assay, CRW significantly increased the activity of SOD in liver, the activities of GSH‐Px and CAT in brain cortex, body quality, thymus index, and spleen index, and decreased liver MDA levels, liver total cholesterol content, and AChE levels in hippocampus. The CRW has potent antifatigue ability and could minimize the occurrence of age‐associated disorders.

## 1 INTRODUCTION

Chinese rice wine (CRW), a traditional Chinese alcoholic beverage, is consumed widely in China and is commonly used as an ingredient in traditional Chinese medicine prescription guiding drug (Li, Shen, & Meng, 2013). Traditional rice wines are made from sticky rice, wheat, and some medicinal plants or herbs fermented by Chinese koji. During its production, starch from rice is converted into alcohol and fermentation by yeast, fungus and lactic acid bacteria. Chinese rice wine contains potentially bioactive components. Fifteen free amino acids and γ‐aminobutyric acid are quantitated by the HPLC‐fluorescence method (Lu et al., 2015). The protein assay demonstrated that CRW is a good source of amino acids (2923 μg/ml), γ‐aminobutyric acid (10.1 μg/ml), and peptides/proteins (4210 μg/ml). Chinese rice wine is also rich in polyphenols, which is mainly from glutinous rice and wheat, including catechin, epicatechin, rutin, quercetin, protocatechuic, chlorogenic acid, caffeic acid, syringic acid, ferulic acid, p‐incense beans, etc. (Bai et al., 2016; Zhen‐Lin, Xia, & Wei‐Min, 2011). The total phenolic content comes to 1.023 mg/ml, which can be comparable with polyphenol content in the red wine (Xie, 2008; Xie, Rong, & Fan, 2011). Although CRW is rich in species and contains high nutrients, the research of physiological function of CRW is still at the early stage (Que, Mao, Zhu, & Xie, 2006).

Fatigue is a complex metabolic phenomenon. During fatigue, body faces difficulty in initiating or sustaining voluntary activities (Ni et al., 2013; Zhang et al., 2010). It may cause various disorders related to bio‐regulatory and immune system. It has been reported that more than 20% of people suffered from chronic fatigue (Nozaki et al., 2009), which is involved in various diseases such as cancer, AIDS Parkinson's disease, and others (Tharakan, Dhanasekaran, & Manyam, 2005). Several studies showed that protein, peptides, and polysaccharide could alleviate fatigue (Ding et al., 2011; Kumar, Chouhan, Sanghi, & Teotia, 2013; Narkhede et al., 2016; Nozaki et al., 2009; Puri, Holmes, & Hamilton, 2004; Zhang et al., 2015). Among all kinds of brewing wines, CRW has the highest amount of protein. The content of amino acid, peptide, and oligopeptide is, respectively, 7.55 times and 4.25 times that in beer. The content of polysaccharides in CRW is much higher than wine and beer. Therefore, we expected that CRW has anti fatigue activities.

Aging is a long and gradual process of functional decline in living organisms (Zhou et al., 1900). Increasing discoveries indicate that oxidative stress plays a crucial role in the process of aging (Nohl, 1993; Singh, Sharad, & Kapur, 2003). Oxidative stress is a result of imbalance between the antioxidant defense system and the formation of reactive oxygen species (Kroker et al., 2012), which has been considered deleterious because of the damage to cell membranes and DNA (Sies, 1992). Although cells and organism are naturally provided with antioxidant systems and nonenzymatic antioxidants that could counteract the potentially injurious oxidizing agents (Fernández‐Tomé et al., 2014), these systems are insufficient to repair oxidative damage entirely (Zheng et al., 2015). The superabundant free radicals may attack histocytes and lead to the severe damage of cell functions and eventually aging and death (Harman, 1992). There is substantial evidence showing that aging is associated with decrease in antioxidant status and age‐dependent increase in lipid peroxidation which closely correlates with diminished antioxidant protection (Schuessel et al., 2006). Polysaccharides, widely distributed in animals, plants, and microorganisms, have been proven to possess potential and potent antioxidant activity (Yu, Duan, Fang, Yan, & Wang, 2009; Zhu et al., 2009). Polyphenolic compounds, in red wine and CRW, play a very important role in the antioxidant function (Fei & Pan, 2006; Fernández‐Pachón, Villaño, GarcíA‐Parrilla, & Troncoso, 2004; Kong, Xie, Hu, Si, & Wang, 2017; Villaño, Fernández‐Pachón, Troncoso, & García‐Parrilla, 2005). Chinese rice wine is rich in polysaccharides and polyphenolic compounds, which is mainly from glutinous rice and wheat. There are few investigations regarding the antiaging effect of CRW up to now.

D‐Galactose‐induced aged animal model is widely used for the study of ageing through oxidative stress (Song, Bao, Li, & Li, 1999). This mimetic aging model was related to the formation of age‐related advanced glycation end products, which can accelerate oxidative tissue damage, abnormal stress, excessive inflammation, and cell apoptosis and affect learning and memory functions(Shoji, Takao, Hattori, & Miyakawa, 2016). In addition, brains and livers of mice are believed to be particularly susceptible to oxidative stress and dysfunction in D‐galactose treatment (Cakatay et al., 2013; Chen, Zhong, Peng, Sun, & Kong, 2010). This work investigates the antifatigue and antiaging properties of CRW in mice, which would provide more clues to explain the healthy functions of CRW.

## 2 MATERIALS AND METHODS

### 2.1 Animals

Forty 12‐week‐age male Kunming mice (28~32 g) were obtained from the Center of Laboratory Animal of Shandong University and maintained in a controlled temperature at 24 ± 2°C with a 12‐hr light/dark cycle and acclimatized for at least 1 week prior to use. Animals were kept in the plastic cages with free access to food and water. All experimental procedures received prior approval from the Animal Use and Care Committee for Research and Education of Shandong Normal University (Jinan, P. R. China).

### 2.2 Wine samples

Chinese rice wine (8% Vol) was provided by millet research institute of Hebei Academy of Agriculture and Forestry Sciences. Chinese rice wine has very complicated process including screening the impurity from millet, immersion, steaming, cooling, adding yeast saccharification yeast to fermentation, pressing, clarification, filtering, and obtaining products.

### 2.3 Chemicals and instruments

All the kits including liver glycogen (LG), blood lactate acid (Fernández‐Tomé et al.), blood urea nitrogen (BUN), superoxide dismutase (SOD) (No.A‐005), malonaldehyde (MDA) (No.A003‐1), total cholesterol (TC) (No.A111‐1), glutathione peroxidase (GSH‐Px) (No.A005), catalase (CAT) (No.A007‐1), and total cholinesterase (AChE) (No.A024) were purchased from Nanjing Jiancheng Bioengineering Institute, Nanjing, China. D‐Galactose was obtained from Solarbio Technology Co. Ltd (Beijing, China). All other chemicals were of analytical grade.

### 2.4 Animal treatment to test antifatigue activity of CRW

A total of 120 8‐week‐age mice were divided into four groups after 1‐week adaptation: A control group was fed with 0.25 ml distilled water by gavage. The three treatment groups were administered with 0.15 ml CRW (low‐dose group), 0.25 ml (middle‐dose group), and 0.4 ml (high‐dose group), respectively. After 2 weeks of treatment, mice were subjected to antifatigue test.

### 2.5 Analysis of Liver glycogen(LG)

Ten mice were randomly taken out from each group. Mice were placed in the swimming pool (100 cm × 50 cm × 40 cm, 25 ± 1.5°C) and were forced to swim without a load 30 min after CRW treatment. After swimming for 90 min, mice were immediately sacrificed and then liver was collected to make into 10% homogenates with normal saline at 4°C as soon as possible. The samples were measured according to the recommended procedures provided by the manufacturer's instructions.

### 2.6 Analysis of Blood lactic acid(BLA)

The forced swimming test (Woenckhaus et al., 2008) was used to evaluate the Levels of BLA. All mice were forced to swim with a load (5% body weight) attached to the tail for 10 min. Blood samples were collected through eye sockets at 0 min and 20 min after swimming. Serum samples were prepared by centrifugation at 1000 g, 4°C for 15 min and then were measured the BLA content using a commercial diagnostic kit provided by Jian Cheng (Jiancheng Diagnostic Systems Inc., Nanjing, People's Republic of China). BLA clearance ratio was calculated as following equation (*a* and *b* representing the BLA concentration of mice 0 min and 20 min after swimming, respectively).

$$\text{BLA clearance ratio}\;=\;\frac{b-a}{a}\times 100$$

### 2.7 Analysis of Blood urea nitrogen(BUN)

Mice were placed in the swimming pool and forced to swim without a load 30 min after CRW treatment. After swimming for 90 min, blood was collected through eye sockets to prepare the serum for use at 0 min and 30 min. The content of BUN was detected by BUN kit provided by Jian Cheng (Jiancheng Diagnostic Systems Inc., Nanjing, People's Republic of China).

### 2.8 Antiaging mice model

Mice were randomly divided into four groups with 10 mice per group: the control group (control) treated with 0.4 ml double distilled water, aging model control group (aging model) treated with 0.4 ml double distilled water, aging model group of CRW 1 (CRW 1) treated with 0.25 ml CRW, aging model group of CRW 2 (CRW 2) treated with 0.4 ml CRW. The mice were intragastrically treated continuously for 56 days once a day. Meantime, aging model control group, aging model group of CRW 1, and aging model group of CRW 2 were administered with a single intraperitoneal injection of D‐galactose (200 mg/kg b.w.) and the control group were injected the same volume of normal saline (Zhou et al., 2013). All mice were fasted for 12 hr before preparing experimental samples, weighed, and recorded.

### 2.9 Determination of the activities of superoxide dismutase (SOD), malondiadehyde (MDA), total cholesterol (TC)

After the mice were sacrificed, the same part of the liver was selected at once, then rinsed with normal saline, and adsorbed the blood by the filter paper. The liver was accurately weighed to 0.1 g, then added normal saline (0.86%) with the proportion of the tissue mass (g): volume (ml) = 1: 9, and centrifuged (1,500 *g*) for 15 min to prepare the serum to detect. The serum samples were measured using commercially available kits according to the manufacturer's instructions. All the treatments were performed at low temperature.

### 2.10 Determination of GSH‐Px and CAT in cerebral cortex and AChE in the hippocampus

After the mice were sacrificed, cerebral cortex and hippocampus were selected and rinsed with normal saline and adsorbed the excess liquid by the filter paper. Cerebral cortex was added normal saline (0.86%) with the proportion of the tissue mass (g): volume (ml) = 1: 9 and centrifuged (1,500 *g*) for 15 min to prepare the serum for determination. The hippocampus was added lysates including protease inhibitors and phosphatase inhibitors with the proportion of the tissue mass (g): volume (ml) = 1:9, homogenized and ultrasonic crushing, placed at 4°C for 30 min, centrifuged (7,200 *g*) for 10 min to prepare the serum for determination. The serum samples were measured using commercially available kits according to the manufacturer's instructions. All the treatments were performed at low temperature.

### 2.11 Detection of body mass and immune organ index in aging mice induced by D‐Galactose

After mice were sacrificed, the thymuses and spleens that removed of lipid and connective tissue surrounding the organs were taken out and adsorbed the blood with the filter paper. The samples were accurately weighted and calculated the index of immune organs and changes the body mass.

$$\text{Immune organ index}\;=\;\frac{\text{Organ weight (mg)}}{\text{Body weight (g)}}$$

### 2.12 Statistical analysis

All the statistics were performed by means of the SPSS version 19.0 software for Windows. All data analysis was tested by one‐way analysis of variance for multiple comparisons and expressed as mean ± *SD*. *p* < 0.05 was considered to indicate the statistically significant.

## 3 RESULTS

### 3.1 LG, BLA, and BUN levels after CRW treatment

**Figure 1:**
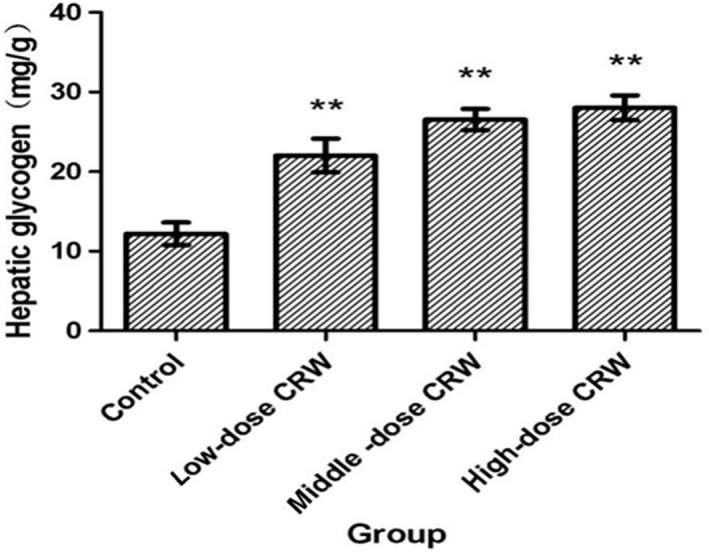
Effects of Chinese rice wine on the contents of liver glycogen (*n* = 10, $\overset{¯}{x}\pm S$ . **p < 0.01 compared with control group

Glycogen content is an integral determining factor in fatigue. Many studies have observed significant depletion of glycogen in both liver and muscles during exhaustive exercise, which is responsible for the fatigue. The liver glycogen levels (LG) were 1.8‐, 2.2‐, and 2.2‐fold higher in low, middle, and high CRW groups, respectively, than that in control group (Figure 1). The data indicated that CRW can significantly increase the content of LG.

BLA is the glycolysis product of carbohydrate under an anaerobic condition. Here, 20‐min rest after swimming, the BLA concentrations were much lower in CRW groups than in the control group (Table 1; *p* < 0.05). The BLA clearance ratio was 1.30%, 7.72%, and 15.90% in low, middle, and high CRW groups, respectively, and significantly increased compared with the control group (−91.18%). The results showed that CRW effectively retarded and lowered the blood lactic acid and increase the body's antifatigue ability.

**Table 1:** Effect of Chinese rice wine on BLA content of mice (*n* = 10, *x* ± *S*, mmol/L)

Groups	0 min after swimming	20 min after swimming	Growth value	Clearance(%)
Control	5.67 ± 1.39	10.85 ± 1.73	5.17 ± 2.13	−91.18 ± 1.06
Low dose	7.46 ± 0.92	7.36 ± 0.63*	−0.1 ± 1.35*	1.30 ± 0.10**
Middle dose	5.31 ± 0.77	4.90 ± 0.43**	−0.41 ± 1.16*	7.72 ± 0.21**
High dose	7.79 ± 1.78	6.55 ± 0.82*	−1.24 ± 1.36*	15.9 ± 0.60**

Compared with the control group, *p < 0.05; **p < 0.01.

Levels of BUN in CRW groups have no significant different (*p* > 0.05) at 0 min and 30 min after exercises compared with the control (Table 2). The growth value and the growth rate of BUN were significantly lower in the high group than that in the control (*p* < 0.05), while the low and middle CRW group was not significantly different to the control (*p* > 0.05; Table 2). The reduced protein metabolism of the high dose of CRW was indicative of enhanced endurance.

**Table 2:** Effect of Chinese rice wine on BUN content of mice (*n* = 10, *x* ± *S*, mmol/L)

Groups	0 min after swimming	30 min after swimming	Growth value	Growth rate (%)
Control	9.78 ± 0.90	14.62 ± 0.80	4.84 ± 0.62	16.13 ± 2.08
Low dose	11.52 ± 0.54	14.81 ± 0.16	3.29 ± 0.67	10.96 ± 2.22
Middle dose	10.75 ± 1.24	14.23 ± 1.10	3.48 ± 0.28	11.59 ± 0.93
High dose	10.62 ± 1.06	13.56 ± 1.52	2.78 ± 0.59*	9.28 ± 1.95*

Compared with control group, *p < 0.05.

### 3.2 Effect of CRW on SOD, MDA, and TC in aged mice

**Figure 2:**
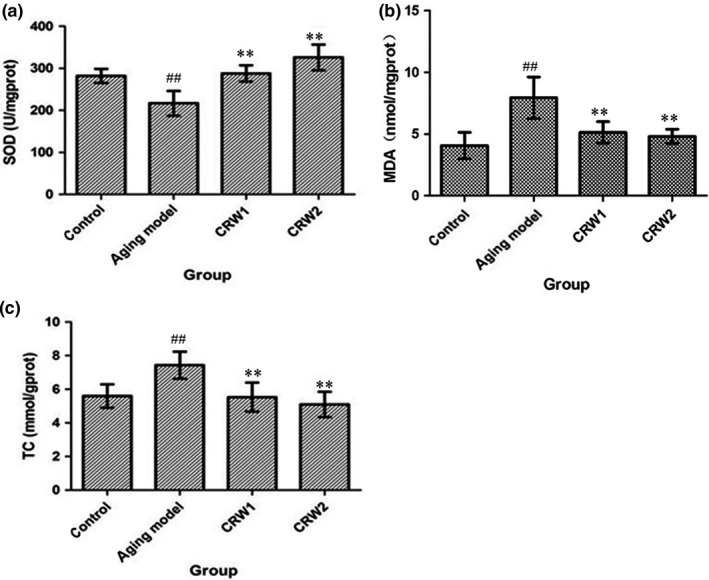
Effects of Chinese rice wine on SOD (a), MDA (b) and TC (c) in liver of D‐Galactose‐induced aged mice (*n* = 10, $\overset{¯}{x}\pm S$ ). ^##^ *p* < 0.01 vs control group; **p < 0.01 vs aging model control group

The content of liver SOD, MDA, and TC among the control group, the aging model group of CRW1, and aging model group of CRW 2, compared with the aging model control group, had statistical significance (*p* < 0.01; Figure 2). Superoxide dismutase activity of the control group, the aging model group of CRW1, and aging model group of CRW 2 was higher than that in aging model control group, while MDA content and TC content were lower than that in the aging model control group (Figure 2). The results showed that CRW could effectively increase the SOD activity and decrease the content of MDA and TC in the liver of subacuted aging mice.

### 3.3 Effect of CRW on GSH‐Px and CAT Activity in aged mice

**Figure 3:**
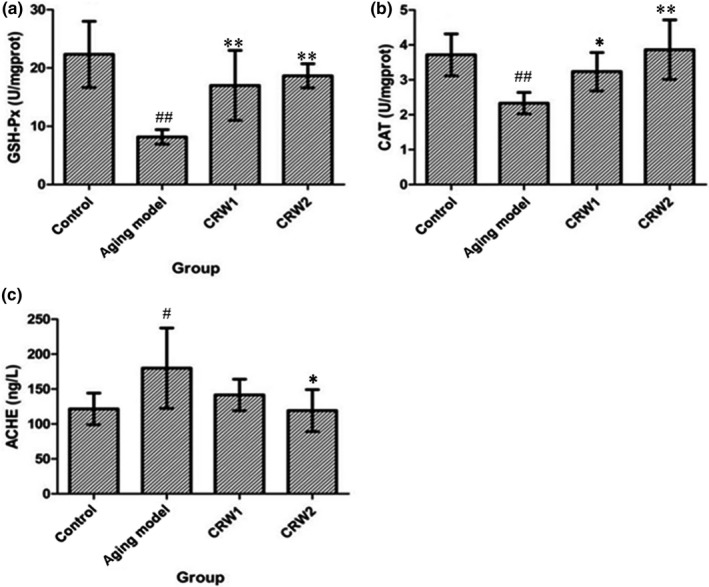
Effects of Chinese rice wine on GSH‐Px (a) and CAT (b) in cerebral cortex and AChE (c) in the hippocampus of D‐galactose‐induced aged mice (*n* = 10, $\overset{¯}{x}\pm S$ ). ^**##**^ *p* < 0.01 vs control group; *p < 0.05, **p < 0.01 vs aging model control group

The activity of GSH‐Px in the cerebral cortex of the control group, aging model group of CRW 1, aging model group of CRW 2 was much higher than that in the aging model control group and had statistical significance (*p* < 0.01)(Figure 3). Compared with the aging model control group, CAT activity in the cortex of the other three groups increased significantly, and the difference among the control group, aging model group of CRW 2 with aging model control group had statistical significance (*p* < 0.01; *p* < 0.05; Figure 3). The hippocampus AChE activities in the control group and aging model group of CRW 2 were obviously lower than that in aging model control group (*p* < 0.01; Figure 3), indicating that CRW could significantly block aging‐induced acetylcholinesterase activity.

### 3.4 Effect of CRW on body mass and immune organ index

There was no difference in the initial body mass and the initial body mass among all groups (Table 3). The body weight growth was 12.99 ± 2.18 g, 9.95 ± 1.11 g, and 10.68 ± 1.10 g in control group, aging model group of CRW1, and aging model group of CRW2, respectively, and significantly increased compared with the aging model control group (*p* < 0.05).

**Table 3:** Effect of Chinese rice wine on the weight of aging mice (*n* = 9, *x* ± *S*, g)

Group	Initial body weight	Finial body weight	Body weight growth
Control	34.30 ± 1.85	46.77 ± 1.87	12.99 ± 2.18**
Aging model control	37.26 ± 1.19	42.68 ± 1.11	5.42 ± 0.86
CRW 1	36.28 ± 0.70	46.23 ± 1.39	9.95 ± 1.11*
CRW 2	36.69 ± 0.82	47.38 ± 1.19*	10.68 ± 1.10*

Compared with aging model control group, *p < 0.05; **p < 0.01.

**Figure 4:**
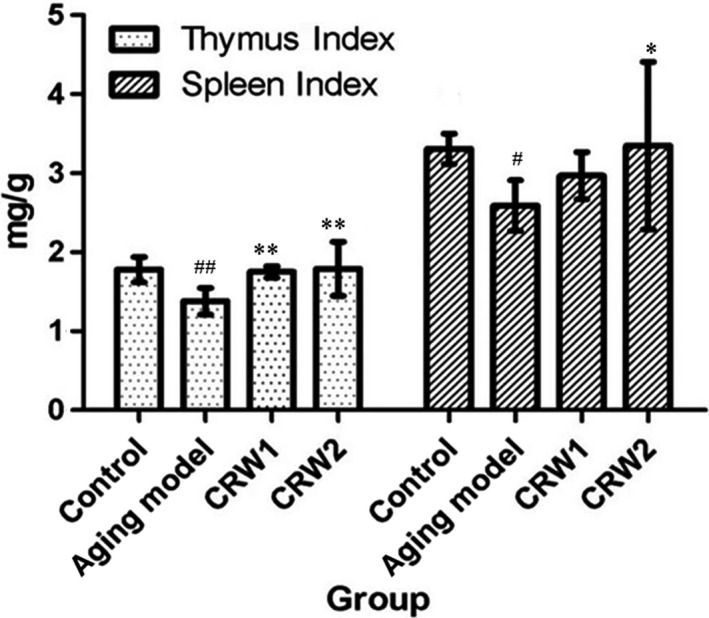
Effect of Chinese rice wine on immune organs (thymus and spleen) index of D‐galactose‐induced aged mice (*n* = 10, $\overset{¯}{x}\pm S$ ). ^#^ *p* < 0.05, ^**##**^ *p* < 0.01 vs control group; *p < 0.05, **p < 0.01 vs aging model control group

As shown in Figure 4, the thymus indexes of mice in control group, aging model group of CRW1, and aging model group of CRW2 were significantly higher than that in aging control group (*p* < 0.01), meanwhile, the spleen index in control and aging model group of CRW2, but not in aging model group of CRW1, also obviously increased in comparative with aging model control group (*p* < 0.05). These results indicated that CRW maybe have positive effects on thymus and spleen of aging mice.

## 4 DISCUSSION

The forced swimming is the most common exercise model to evaluate the antifatigue effect of test compounds (Ding et al., 2011; You, Ren, Yang, Regenstein, & Zhao, 2012). Glycogen is a polysaccharide structure, which was the important resource of energy during exercise. Glycogen was used for long‐term energy storage and could be to complement the consumption of blood glucose and maintain the blood glucose in the physiologic range (Wang & An, 2011). Fatigue will happen when the glycogen is mostly consumed. So glycogen is a sensitive index to test fatigue (You, Zhao, Regenstein, & Ren, 2011). In our study, LG levels in CRW groups were significantly higher than that in control group (*p* < 0.05), indicating that CRW can significantly increase the content of LG after swimming. Yellow rice wine contains a large number of carbohydrates which are in the form of monosaccharide and can be directly absorbed, metabolism or turned to glycogen stored in liver and muscle to meet the energy needs of tissues (Nakatani et al., 1997). In addition, the abundant oligosaccharides and active peptides in CRW can improve the antifatigue ability of mice by increasing the oxygen‐resistant capacity of cells and the ability of muscle contraction and by promoting the repair of muscle cells during exercise and after exercise (Guo et al., 2015; Wang, Shieh, Kuo, Lee, & Pan, 2006). The increasing levels of BLA will decrease internal pH value in muscle tissue and blood, which could lead to fatigue (Lu et al., 2015). BUN is the metabolism outcome of protein and amino acid. Protein and amino acids have a stronger catabolic metabolism when body cannot obtain enough energy by sugar and fat catabolic metabolism. The catabolism of protein and amino acid will be strengthened along with the increase of training intensity, which contribute to increase the level of BUN (You et al., 2011). Our results showed that CRW can significantly decrease the levels of BLA and BUN after swimming, which suggested the antifatigue effect of CRW. The mechanism may be related to polyphenols in rice wine, which can improve the activity of lactate dehydrogenase and mitochondrial antioxidant enzymes, and has regulated and protected function to mitochondria (Wang, Zhao, & Zhang, 2015).

Free radical theory strengthens the hypothesis that aging occurs by the accumulation of oxidative damage, such as DNA mutation and lipid and protein dysfunction (Song et al., 1999). CRW has strong antioxidant capacity, and it can be used as the important natural antioxidants (Aydin et al., 2012). Oversupply of D‐galactose could induce accumulation of ROS or stimulate free radical production indirectly through oxidative metabolism of it and glycation end products to accelerate aging. Therefore, D‐Galactose‐induced aged animal model is widely used for the study of ageing through oxidative stress. Brains and livers of mice are considered to be particularly susceptible to oxidative stress and dysfunction in D‐Galactose treatment (Harman, 2009; Liochev, 2015). Superoxide dismutase is an important member of antioxidant enzymes, from humans to lower animals, to plants, to single‐celled microorganisms, all exists. It exerts the antioxidant activity and eliminates oxidative damage to the cell (Meeusen & De Pauw, 2015; Morgan, Costill, Flynn, Raglin, & O'Connor, 1988). Due to its strong antioxidant capacity, it can prevent and treat a variety of diseases caused by the oxide free radicals (Smith & Doolittle, 1992). Malondialdehyde (MDA) is the final product of lipid peroxidation reaction caused by free radicals produced through the enzyme system and non‐enzymatic system and then acted on unsaturated fatty acids of the biomembrane. The detection of MDA provides a convenient tool for quantitative determination of membrane lipid peroxidation, which indicates the damage to the membrane system and oxidative damage of the cell (Auer et al., 1995; Gunes et al., 2016). The total cholesterol (TC) level is associated with aging (Tukiainen et al., 2012). In agreement with previous studies used similar drinks, our study demonstrated that treatment with CRW significantly reduced MDA, TC and improved SOD activity in livers of D‐gal‐induced ageing treated groups when compared to the ageing untreated group.

Chinese rice wine is produced from glutinous rice. During its production, starch from rice is converted into alcohol and fermentation by yeast, fungus, and lactic acid bacteria (Deng et al., 2016). Chinese rice wine also contains potentially bioactive components. Fifteen free amino acids and γ‐aminobutyric acid were quantitated by the HPLC‐fluorescence method. The protein assay demonstrates that CRW is a good source of amino acids (2923 μg/ml), γ‐aminobutyric acid (10.1 ± 0.3 μg/ml), and peptides/proteins (4210 ± 430 μg/ml) (Lu et al., 2015). The total phenolic content comes to 1.023 mg/ml, which can be comparable with polyphenol content in the red wine (Fei & Pan, 2006; Xie, 2008; Xie et al., 2011). A number of studies have shown that CRW exhibited high antioxidant power and that total antioxidant activity, reducing capacity, and free radical scavenging activity were highly correlated with total phenolic content (Que et al., 2006). At the same time, the polysaccharides from CRW at 1.0 mg/ml scavenged the DPPH, hydrogen peroxide, superoxide ion at 92%, 53%, and 86%, respectively (Peng et al., 2012). Therefore, it is possible that antiaging effect of CRW on aging mice is due to antioxidant properties of polyphenol and polysaccharide in CRW.

For dealing with reactive byproducts of the metabolic process, higher eukaryotes have developed antioxidatant defense strategies, including enzymatic systems, such as CAT and GSH‐Px, to protect cells from oxidative damages (Rocha, Gomes, Lima, Bronze‐da‐Rocha, & Santos‐Silva, 2015; Sin et al., 2013). CAT occurs in almost all biological cells, while GSH‐Px is especially important for brain. CAT and GSH‐Px catalyze the dismutation of H2O2 to H2O and O2. In addition, we measured the content of AChE in hippocampus. It postulates that the impairment of the memory results from the low levels of the neurotransmitter, Acetylcholine (ACh). AChE is the hydrolytic cholinesterase enzymes that act on ACh at the synaptic cleft to terminate the cholinergenic signal transfer (Kroker et al., 2012). Therefore, prolonging the half‐life of Ach by the use of cholinesterase inhibitors which are responsible for the hydrolysis of AChE and terminating its function is currently used as a treatment approach of neurological disorders. Our results showed that the CAT, GSH‐Px activities in cerebral cortices were improved and AChE activity in hippocampus was greatly inhibited in ageing mice treated with CRW. The observed activities might be due to the ability of phenolic compounds, such as phenolic acids, flavonoids, and quinones in CRW.

Body mass is the index of evaluating animals growth and reflecting body synthesis function(Liu, Xie, & Zhou, 2018). Thymus and spleen as the central and important peripheral immune organs, its atrophy, decreased quality, functional decline can reduce immune function, so the thymus index and spleen index are used to check the immune organ growth and immune cell function indicators (Fan, Ding, Ai, & Deng, 2012; Wang et al., 2012; Yang, Jia, Zhou, Pan, & Mei, 2012). In this study, body mass, thymus index, and spleen index in mice treated with CRW were significantly higher compared with age control group without CRW treatment, indicating that CRW had promoting effect on the immune organ index of aging mice induced by D‐Galactose.

## 5 CONCLUSION

This study presents that CRW could increase the content of liver glycogen, SOD in liver, GSH‐Px and CAT in brain cortex, body quality, thymus index, and spleen index, and decrease the levels of BLA and BUN, liver MDA levels, liver total cholesterol content, and AChE levels in hippocampus. The study shows that CRW has antifatigue ability and could minimize the occurrence of age‐associated disorders, which provides more clues of CRW healthy functions.

## CONFLICT OF INTERESTS

The authors declare that they do not have any conflict of interest.

## ETHICAL REVIEW

This study was approved by the Animal Use and Care Committee for Research and Education of Shandong Normal University (Jinan, P. R. China).
